# ViVo: A Temporal Modeling Framework That Boosts Statistical Power and Minimizes Animal Usage

**DOI:** 10.1021/acs.jmedchem.5c03326

**Published:** 2025-12-29

**Authors:** Guillermo Canudo-Barreras, Eduardo Romanos, Raquel P. Herrera, M. Concepción Gimeno

**Affiliations:** † Instituto de Síntesis Química y Catálisis Homogénea (ISQCH) CSIC-Universidad de Zaragoza, C/Pedro Cerbuna 12, Zaragoza 50009, Spain; ‡ Servicio de Imagen Medica y Fenotipado, 59550 Instituto Aragonés de Ciencias de la Salud, Centro de Investigación Biomédica de Aragón (CIBA), Avda. San Juan Bosco, 13, Planta D, Zaragoza E-50009, Spain

## Abstract

Preclinical tumor studies are often limited by high variability and small-sample sizes, reducing statistical power, and masking treatment effects. We present an exponential framework that estimates tumor growth rates (*r*) independently of initial burden and introduces Tumor Growth Rate (TGR) matrices to map treatment effects across time windows. Applied to a public xenograft data set and four additional mouse models, exponential fits achieved high agreement with raw data (median *R* ^2^ = 0.937). When resampling matched group sizes (*n* = 3–7), our approach consistently outperformed conventional end point and daily nonparametric analyses, revealing treatment effects that standard comparisons missed. The framework also predicts tumor weights for animals euthanized early, enabling their inclusion in final analyses and enhancing statistical consistency while advancing the 3Rs principles. To ensure broad adoption, we developed **ViVo**, an open-source web platform (gcanudo-barreras.github.io/ViVo-Platform/) that provides accessible, standardized analysis of in vivo tumor kinetics and therapeutic efficacy.

## Graphical abstract

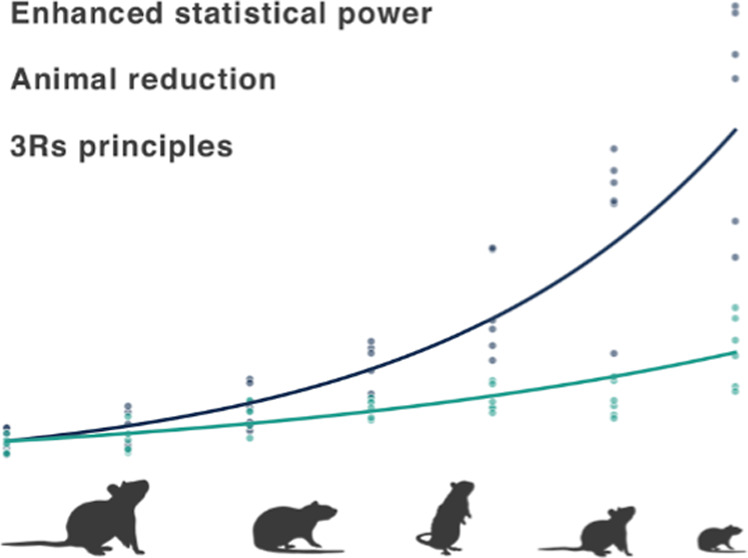

## Introduction

Preclinical oncology faces a critical challenge: while computational advances have revolutionized drug discovery through machine learning^1,2^ and sophisticated algorithms,^3^ practical implementation remains limited by analytical complexity and the need for extensive animal cohorts. Most preclinical studies rely on oversimplified tumor comparisons using classical statistics with insufficient sample sizes, a limitation that contributes to the low success rate, with only 12–36% of compounds showing efficacy in animal models ultimately advancing to Phase III clinical trials.^4^

This analytical gap is evident in recent studies. Meng, Zhang, and co-workers reported critical inconsistencies in which tumor weights revealed significant treatment differences that were not detected by volume measurements (Figure 1A).^5^ Similar discrepancies were noted by Rong, He, and co-workers, further undermined by statistical flaws such as applying one-way ANOVA to longitudinal tumor growth data.^6^ Kolesar and Awuah conducted an efficacy study with only four mice per group, analyzing the data using a Student’s *t*-test, a method with limited robustness for such small-sample sizes.^7^ Even bioluminescence imaging faces interpretation difficulties, with different visualization techniques yielding distinct results, thereby compromising data reliability (Figure 1B).^8^

**Figure 1:**
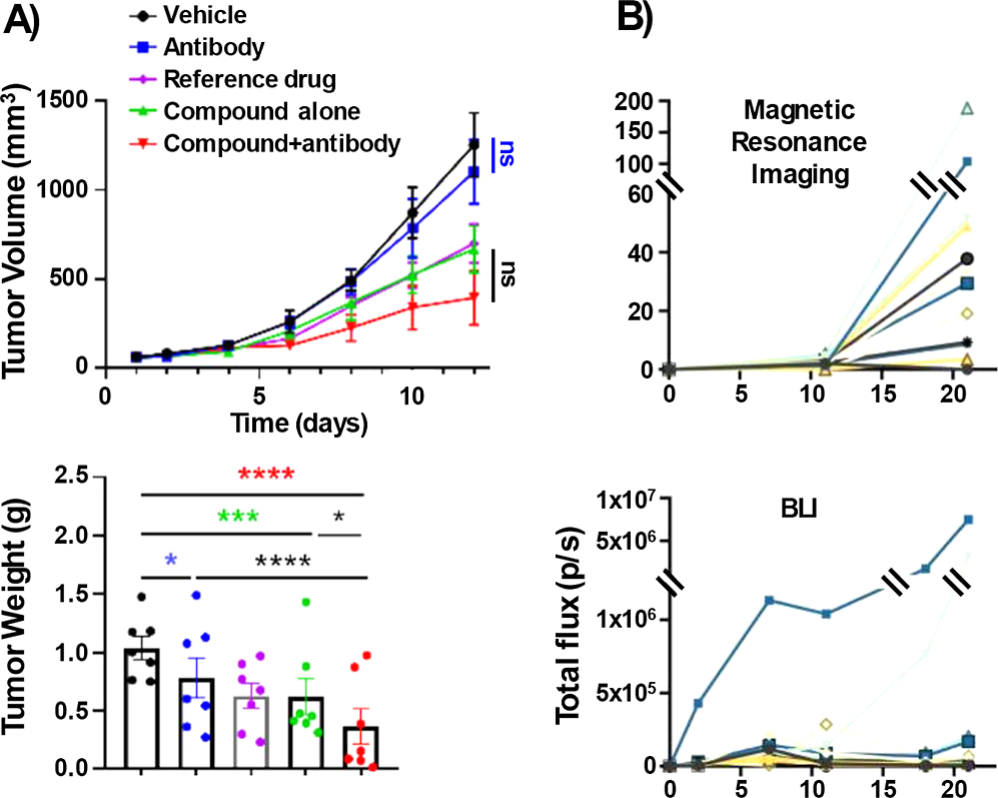
Current methodological limitations in preclinical oncology demonstrate systematic inconsistencies between measurement approaches and inadequate statistical power with small-sample sizes. (A) ENPP1 inhibitor study (Meng, Zhang)-weight vs volume discrepancies. Adapted from ref 5. Copyright 2025 American Chemical Society.^5^ (B) BLI instability in glioblastoma. Adapted from ref 8. Available under a CC BY 4.0 license. Copyright 2023 Préat, Gallez.^8^

These methodological inconsistencies could theoretically be mitigated by increasing sample sizes, but current research practice is constrained by ethical imperatives that explicitly discourage such approaches.^9^

The internationally recognized 3Rs framework, Replacement, Reduction, and Refinement, promotes minimizing animal usage while maximizing data quality.^10^ In parallel, compliance with ARRIVE 2.0 guidelines mandates rigorous study design, transparent reporting, and statistically justified animal numbers.^11^ These principles present a practical and ethical responsibility: to extract richer, more informative insights from smaller cohorts rather than rely on numerical expansion to compensate for analytical limitations. The field requires methodological strategies that enhance interpretability and statistical power without increasing the animal burden.

To address this ethical-methodological gap, we propose an exponential modeling solution (eq 1)­

$$N\left(t\right)=N_{0}e^{rt}$$ 1

where *N*(*t*) represents the number of tumor cells at time *t*, *N*_0_ is the initial tumor burden, and *r* defines the growth rate (time^–1^). This approach enables a fundamental shift from snapshot-based measurements (*N* comparison) to pattern-based analysis (*r* comparison).

The exponential model has proven effective across multiple studies.^12,13^ Both tumor volume and bioluminescence exhibit an approximately linear dependence on cell number, making them suitable parameters for this type of modeling, despite their apparent complexity. Two key assumptions underlie the model: cells divide continuously and without constraint. Its application is, therefore, most appropriate during early stage tumor growth,^14^ before angiogenesis and nutrient depletion reshape proliferation dynamics.^15^

At the core of our approach are TGR matrices, analytical tools that capture temporal treatment dynamics, exposing growth patterns and trends that remain hidden in end point analyses. Beyond the descriptive value, time-resolved modeling of tumor size provides predictive capabilities, such as estimating tumor weight on defined days relative to sacrifice. This enables standardized tumor weight comparisons across animals within a study, even when euthanasia occurs at different times due to humane end point criteria.

These methodologies are implemented in the ViVo platform, an accessible web-based tool for systematic tumor growth analysis (Figure 2). Beyond enabling exponential modeling, TGR matrices, and time-resolved tumor weight standardization, ViVo also facilitates the systematic identification and removal of outliers and includes an advisory tool to assess animal model homogeneity, providing researchers with a standardized approach to improve data consistency and interpretability without increasing cohort sizes. Together, these features allow more reliable, ethically responsible analysis of preclinical tumor studies, extracting richer insights from limited animal data sets.

**Figure 2:**
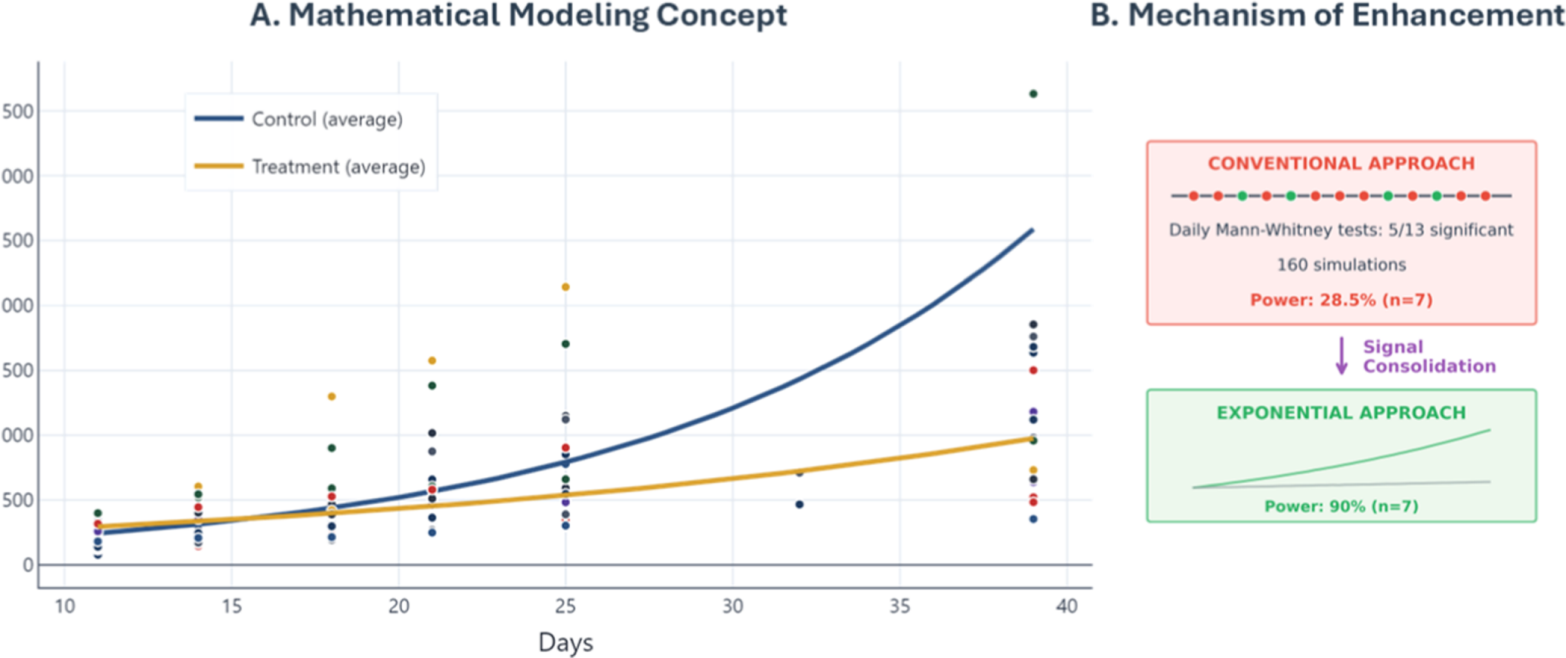
Schematic representation of the ViVo platform, which applies exponential modeling and temporal analysis to convert conventional tumor measurements into robust growth parameters.

## Results

### Enhanced Statistical Power with Reduced Sample Size Requirements

Exponential modeling demonstrated superior statistical power across all tested sample sizes, maintaining performance levels that are unattainable through conventional analysis. To confirm model applicability and assess its statistical power, we used the publicly available data set of Dr. Constantine Daskalakis (https://www.causeweb.org/tshs/tumor-growth/), derived from a subcutaneous xenograft mouse model with human glioma cells consisting of 18 animals (8 control and 10 treated mice, *n* = 8,10) with 13 time points (days 0, 3, 4, 5, 6, 7, 10, 11, 12, 13, 14, 17, and 18).^16^

Mathematical modeling achieved exceptional goodness-of-fit across the data set. 17/18 animals achieved coefficient of determination *R* ^2^ > 0.8 ( $\overset{®}{R^{2}\;}$ = 0.9440 for control group, $\overset{®}{R^{2}\;}$ = 0.9166 for drug group), while 13/18 reached *R* ^2^ > 0.9 ( $\overset{®}{R^{2}\;}$ = 0.9523 for control group, $\overset{®}{R^{2}\;}$ = 0.9456 for drug group). With *R* ^2^ > 0.8 filtering, exponential modeling revealed a 31.9% reduction in growth rate between treated and control groups (*r* = 0.175 ± 6.9% vs 0.257 ± 11.3% day^–1^, respectively). Analogously, *R* ^2^ > 0.9 filtering achieved a 29.8% reduction in growth (*r* = 0.193 ± 5.8% vs 0.274 ± 10.8% day^–1^; Supporting Information Table S1).

Systematic evaluation across eight sample size scenarios (from *n* = 10,8 down to *n* = 3,3 per group) revealed the exceptional performance of the framework in limited-sample contexts (Table 1).

**Table 1:** Statistical Power Comparison Using a Daskalakis Public Dataset.^16^ ^a^

entry	sample size *n* per group	conventional analysis statistical power (%)	exponential model		improvement vs conventional
			*R* ^2^ > 0.8	*R* ^2^ > 0.9	
1	*n* = 10, *n* = 8	61.5	**100**	**100**	**+38.5%**
2	*n* = 9, *n* = 8	53.1	**100**	**100**	**+46.9%**
3	*n* = 8, *n* = 8	41.9	**100**	**100**	**+58.1%**
4	*n* = 7, *n* = 7	28.5	**40**	**90**	**+61.5%**
5	*n* = 6, *n* = 6	22.3	**30**	**70**	**+47.7%**
6	*n* = 5, *n* = 5	9.7	**40**	**40**	**+30.3%**
7	*n* = 4, *n* = 4	7.0	**20**	**35** ^a^	**+28%**
8	*n* = 3, *n* = 3	0.0	**35**	**40** ^a^	**+40%**

a For extremely small samples (*n* ≤ 4), subsets with insufficient animals after quality filtering were excluded from power calculations.

Mathematical modeling demonstrated superior statistical power across all configurations, maintaining 100% performance down to *n* = 8,8 groups (Table 1, entries 1–3) and 90% power with *n* = 7,7 samples (Table 1, entry 4). Even at extreme sample sizes (*n* = 3,3), exponential modeling achieved 35–40% power, compared to 0% for conventional analysis (Table 1, entry 8). Conventional analysis employed daily Mann–Whitney *U* tests with statistical power calculated as the percentage of days achieving significance, reaching a maximum of 61.5% even with the largest cohort tested (Table 1, entry 1).

This enhancement is visualized across the complete sample size range (Figure 3), where exponential modeling maintains a robust performance even with extremely limited cohorts.

**Figure 3:**
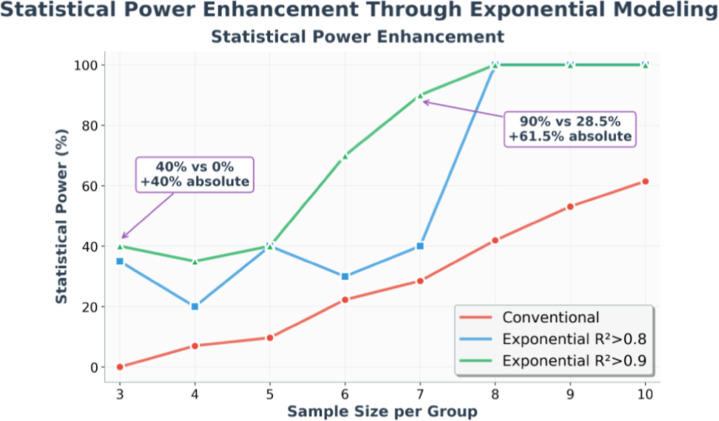
Exponential modeling enhances statistical power across all sample sizes. Statistical power comparison using Daskalakis data set: conventional analysis (red circles), exponential modeling with quality filters *R* ^2^ > 0.8 (blue squares) and *R* ^2^ > 0.9 (green triangles).

The mechanistic basis for this improvement arises from fundamental principles of error propagation (Supporting Information Note S1). Analysis of parameter variability demonstrates a systematic advantage of evaluating growth rates over tumor volume measurements. Specifically, the relative standard error (RSE) for growth rates is consistently lower: RSE­(*r*) = 9.1% *versus* RSE­(*V*_0_) = 17.6%, corresponding to a 48% reduction in parameter uncertainty. In exponential growth models, the uncertainty in the growth rate scales quadratically with time, whereas uncertainty in the initial tumor burden contributes linearly to measurement variability (eq 2).

2 $$RSE\left(N\right)=\surd \left[RSE^{2}\left(N_{0}\right)+t^{2}\cdot RSE^{2}\left(r\right)\right]$$

Consequently, using growth rates instead of raw biomarker data for comparison reduces variability, while enabling robust long-term analysis.

### Temporal Pattern Discovery through TGR Matrix Analysis

While exponential modeling demonstrated superior statistical power by extracting single growth parameters from entire longitudinal studies, systematic application of Tumor Growth Rate matrices revealed that treatment efficacy exhibits distinct temporal heterogeneity invisible to conventional end point analysis. Real therapeutic interventions often exhibit temporal dynamics: drug bioavailability fluctuates, resistance mechanisms emerge, and cellular vulnerabilities shift over time; creating treatment dynamics that single-parameter models cannot detect.

To capture this temporal complexity while keeping the mathematical rigor, we extended the exponential modeling principle to localized temporal intervals. Rather than calculating one growth rate for the entire study period, we systematically calculated growth rates between two time points *x* and *y* (eq 3)­

3 $${r_{x}}_{\to y}=\frac{\ln\left(N_{y}/N_{x}\right)}{t_{y}-t_{x}}$$

This localized growth rate (*r*_*x*→*y*_) represents the instantaneous growth dynamics between specific time points.

When applied to the Daskalakis data set, TGR analysis generated 78 distinct temporal intervals (all possible day-to-day combinations; Figure 4A,B), compared to 13 single time points in conventional analysis, providing 6-fold higher resolution. This enhanced resolution revealed three main tumor growth phases with distinct therapeutic characteristics.

**Figure 4:**
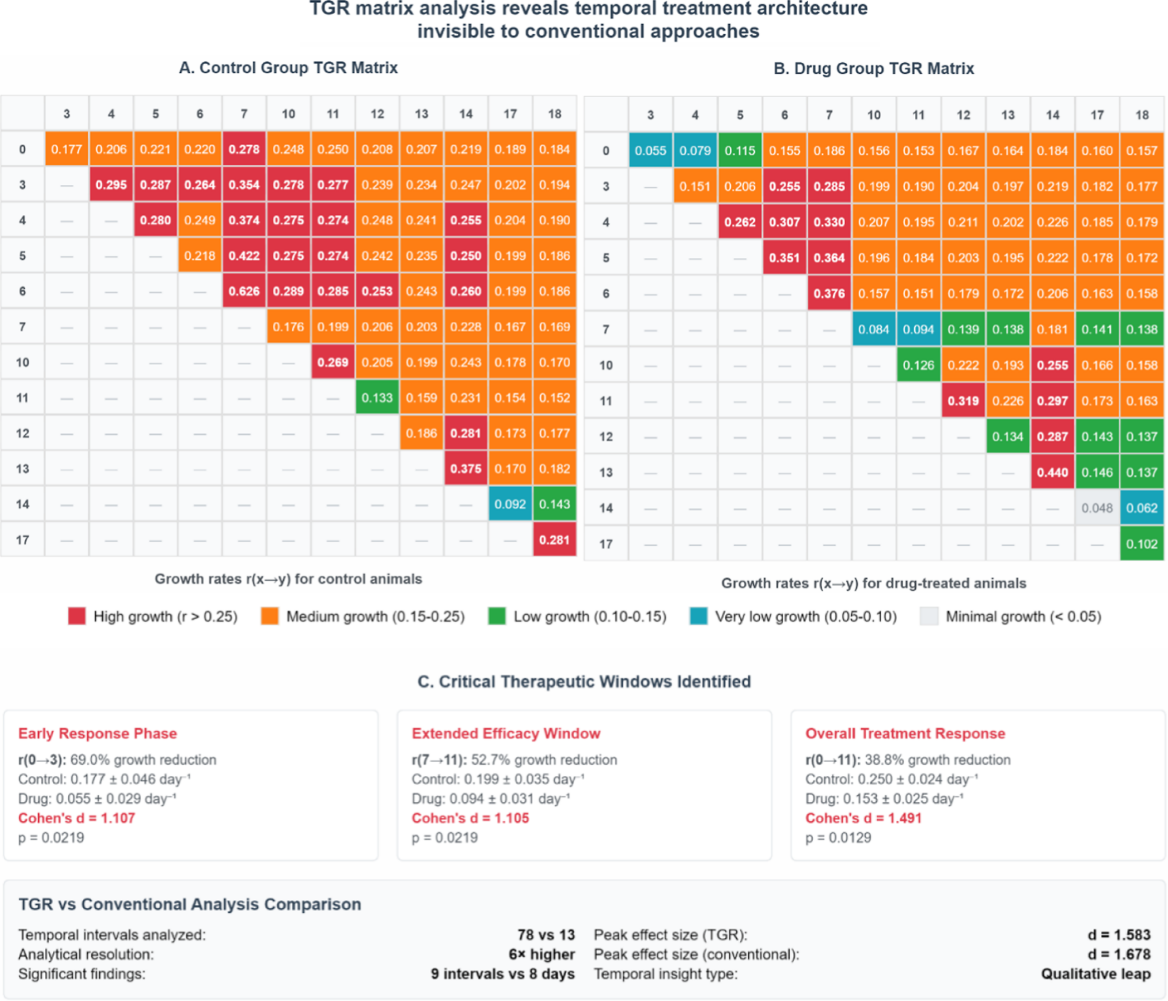
Temporal tumor growth rate (TGR) matrix analysis. (A,B) TGR matrices for control (A) and drug-treated (B) groups showing growth rates (day^–1^) between all time point pairs from day 0 to 18. Color intensity reflects growth rate magnitude: red indicates high growth rates (*r* > 0.25), orange indicates medium rates (*r* = 0.15–0.25), green indicates low rates (*r* = 0.10–0.15), blue indicates very low rates (*r* = 0.05–0.10), and pale gray indicates minimal growth, zero or negative rates (*r* < 0.05). (C) Statistical summary of the three tumor growth phases identified through systematic TGR matrix analysis.

Early response (*r*_0→3_) demonstrated the most pronounced effect, with 69.0% growth rate reduction observed in the first 3 days (0.055 ± 0.029 vs 0.177 ± 0.046 day^–1^ control, p-value = 0.0219, Cohen’s *d* = 1.107).

Extended efficacy (*r*_7→11_) showed 52.7% sustained suppression during midtreatment phase (0.094 ± 0.031 vs 0.199 ± 0.035 day^–1^ control, p-value = 0.0219, Cohen’s *d* = 1.105), from day 7 to 11.

Long-term response (*r*_0→11_) maintained 38.8% comprehensive efficacy (0.153 ± 0.025 vs 0.250 ± 0.024 day^–1^ control, p-value = 0.0129, Cohen’s *d* = 1.491) after 11 days of treatment.

While traditional statistical comparisons identified differences on 8 of 13 study days (Supporting Information Table S2), TGR analysis identified significant treatment effects in 9 of 78 intervals (11.5%) with effect sizes ranging from Cohen’s *d* = 1.105 to *d* = 1.583 (Supporting Information Note S2). Multiday intervals consistently achieved large effect sizes (Cohen’s *d* > 1.1), with peak efficacy during the *r*_0→3_ window, followed by the *r*_7→11_ phase. These systematic temporal patterns demonstrate heterogeneity that is invisible to single-day measurements and undetectable by traditional statistical approaches (Figure 4C), uncovering therapeutic dynamics that could guide the design of combination therapies.

### Cross-Platform Validation and Predictive Applications

The framework demonstrated robust performance across four additional experimental models: three orthotopic tumor models in athymic nude mice using both human and murine cell lines with different kinetic profiles plus one subcutaneous syngeneic model in immunocompetent mice. Animal models included: **(1)** syngeneic orthotopic mammary gland adenocarcinoma using 4T1-Luc2 cells in 16 athymic mice (*n* = 8,8); **(2)** xenogeneic orthotopic triple-negative breast cancer using MDA-MB-231-Luc2-GFP human cells in 12 athymic nude mice (*n* = 6,6); **(3)** syngeneic orthotopic pancreatic ductal adenocarcinoma (PDAC) using MLK 3287-Luc cells in 16 athymic nude mice (*n* = 8,8); and **(4)** subcutaneous syngeneic breast model using 4T1-Luc2 cells in 21 immunocompetent BALB/cByJRjmice (*n* = 10,11), with data from our previous study “Synthetic ease and exceptional in vivo performance of pyrazole-based cyclometalated iridium complexes”.^17^

After applying *R* ^2^ > 0.8 filtering, mathematical modeling proved successful across all experimental groups, with *R* ^2^ values between 0.8683 and 0.9586, and valid animal percentages above 67% in all cases (Table 2).

**Table 2:** Exponential Fitting Metrics to the Used Mouse Models Data

entry	mouse model	group	*N*_0_	*r* (day^–1^)	*R* ^2^	valid animals
1	orthotopic 4T1-Luc2^a^	control	2.99 × 10^8^ ± **10.6%**	0.670 ± **4.7%**	**0.9357**	7/8
2		treatment	2.94 × 10^8^ ± **24.9%**	0.534 ± **9.1%**	**0.9192**	6/8
3	Xenogeneic MDA-MB-231-Luc2-GFP^b^	control^c^	5.4 ± **60.1%**	0.135 ± **19.7%**	**0.8683**	4/6
4		treatment	4.9 ± **43.4%**	0.146 ± **13.0%**	**0.9500**	6/6
5	orthotopic MLK 3287-Luc^a^	control	7.69 × 10^7^ ± **37.6%**	0.232 ± **8.4%**	**0.9092**	7/10
6		treatment	1.05 × 10^8^ ± **24.4%**	0.177 ± **8.9%**	**0.8928**	8/10
7	subcutaneous 4T1-Luc2^b^	control	74.2 ± **17.9%**	0.107 ± **6.1%**	**0.9586**	10/10
8		treatment	152.0 ± **16.6%**	0.054 ± **11.9%**	**0.9475**	8/11

a Tumor size was estimated by BLI, then *N*_0_ = *BLI*_0_ (p/s).

b Tumor size was estimated by caliper, then *N*_0_ = *V*_0_ (mm^3^).

c *R* ^2^ threshold lowered to > 0.7 due to extremely small-sample size (*n* = 3 valid animals with *R* ^2^ > 0.8).

Relative standard errors for the initial tumor burden were consistently higher than those for growth rates (RSE­(*N*_0_) > RSE­(*r*)). Growth rate analysis revealed substantial kinetic differences between cell lines: 4T1-Luc2 cells exhibited *r* = 0.670 day^–1^ compared to *r* = 0.135 day^–1^ for MDA-MB-231-Luc2-GFP cells.

Once mathematical modeling proved successful in describing all of the models, growth rates were compared between experimental groups. The results demonstrated both concordance with conventional analysis and enhanced detection capabilities:

Positive Control Validation: Traditional statistical comparison of orthotopic 4T1-Luc2 model data (Supporting Information Table S3) revealed significant differences on days 4 (p-value = 0.015) and 7 (p-value < 0.001), with corresponding growth rate differences (p-value = 0.012, Cohen’s *d* = 1.341). Conventional statistical analysis of subcutaneous 4T1-Luc2 model data (Supporting Information Table S4) also showed significant differences on days 21 (p-value = 0.045, Cohen’s *d* = 1.194) and 32 (p-value = 0.005, Cohen’s *d* = 1.381), with tumor growth differences (p-value < 0.001, Cohen’s *d* = 2.736).

Negative Control Validation: No significant treatment effects were observed for the xenogeneic orthotopic MDA-MB-231-Luc2-GFP mouse model in either conventional analysis (Supporting Information Table S5) or growth rate comparisons (p-value = 0.366, Cohen’s *d* = 0.348).

Enhanced Detection Capability: While conventional analysis detected no significant differences across study duration for the orthotopic MLK 3287-Luc animal model (Supporting Information Table S6), mathematical modeling and tumor growth comparison revealed significant rate differences (p-value = 0.043, Cohen’s *d* = 1.146).

TGR analysis of each model revealed distinct efficacy phases across the different studies:

Syngeneic Orthotopic 4T1-Luc2: The control group maintained sustained high growth rates throughout the study, while the treatment group showed significantly lower growth rates from day 4 to 7 (*r*_4→7_: 0.502 vs 0.756 day^–1^ control; Figure 5A), representing a 33.6% growth reduction (p-value = 0.007, Cohen’s *d* = 1.471).

**Figure 5:**
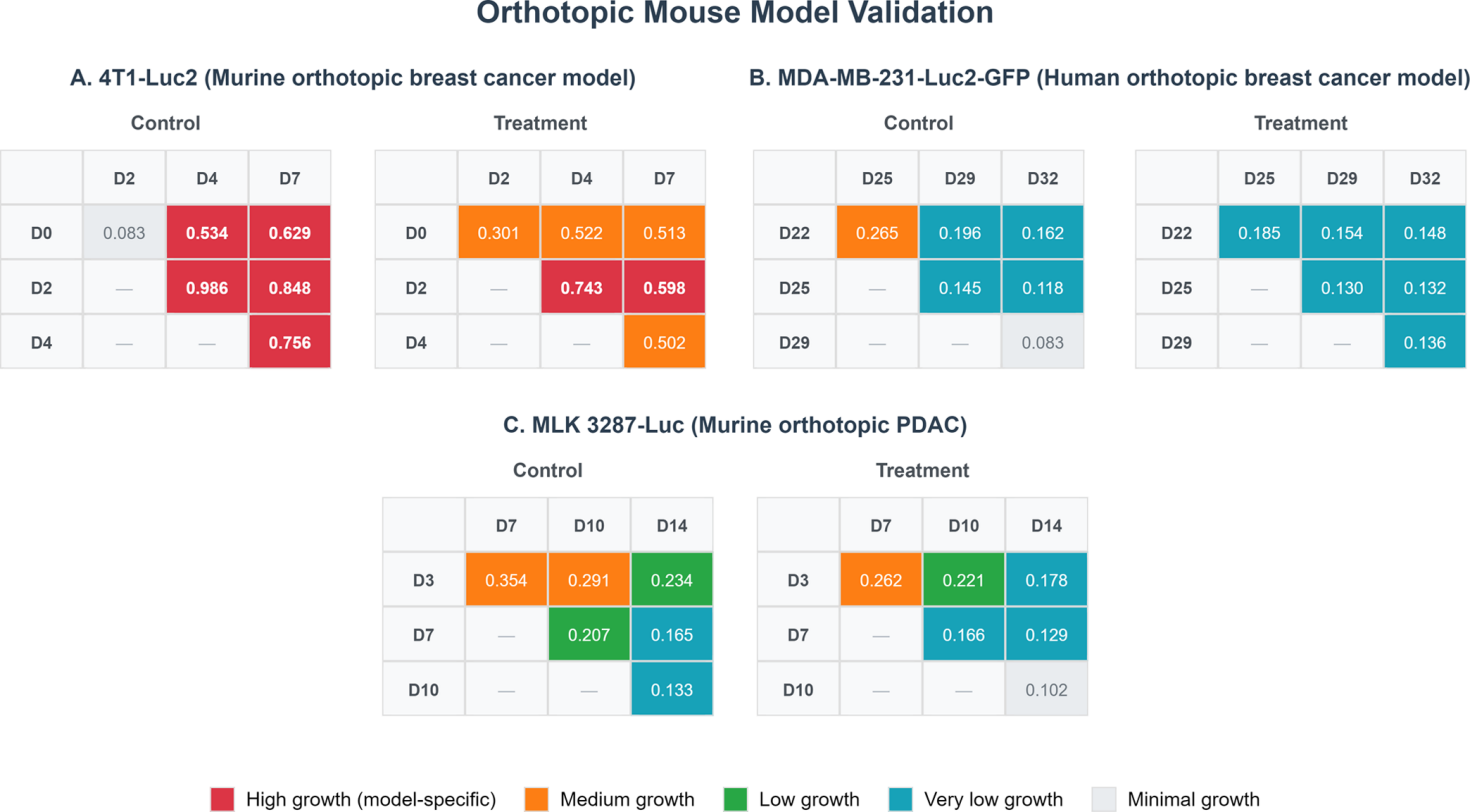
Validation of orthotopic mouse models through TGR analysis. (A) TGR matrices for the murine breast 4T1-Luc2 model (days 0–7). (B) TGR matrices for the human triple-negative breast MDA-MB-231-Luc2-GFP model (days 22–25). (C) TGR matrices for the murine PDAC MLK 3287-Luc model (days 3–14).

Xenogeneic Orthotopic MDA-MB-231-Luc2-GFP: Both groups exhibited similar growth rates across all TGR phases with no significant differences between control and treatment groups in any interval (Figure 5B).

Syngeneic Orthotopic MLK 3287-Luc: Control and treatment groups showed longitudinal deceleration over time (control: *r*_3→7_ = 0.354 day^–1^ → *r*_10→14_ = 0.133 day^–1^; treatment: *r*_3→7_ = 0.282 day^–1^ → *r*_10→14_ = 0.102 day^–1^), evidencing a distinct kinetic profile compared to previous models. Nonetheless, the treatment group experienced greater decline with significant differences emerging in the final study interval (*r*_3→14_: 0.178 vs 0.234 day^–1^ control; p-value = 0.049; Cohen’s *d* = 1.245; Figure 5C).

Subcutaneous Syngeneic 4T1-Luc2: From day 11, the treated group maintained consistently lower growth rates than the control, with peak 67.8% reduction from day 14 to 21 (*r*_14→21_: 0.039 vs 0.122 day^–1^ control). Only one animal in the control group reached day 39. Hence, statistical comparisons between TGR intervals that involved day 39 were not possible. For the 15 valid time intervals, significant differences were observed for 12 of them (Supporting Information Table S7).

Mathematical modeling enabled accurate prediction of tumor weights across different euthanasia dates, addressing the practical challenge of humane end point criteria. Exponential fitting for the subcutaneous 4T1-Luc2 model yielded $\overset{®}{R^{2}\;}$ = 0.9586 for the control group and $\overset{®}{R^{2}\;}$ = 0.9475 for the treated group (Table 2, entries 7 and 8). Direct validation on day 39 was possible for five animals for which both predicted and experimental tumor volumes were available, allowing estimation of the prediction error. Comparison between experimental and predicted values demonstrated prediction errors ranging from 1.2% to 40.6%, with most estimates within 11% accuracy (Table 3).

**Table 3:** Tumor Weight Estimations on Day 39 with Caliper-Based Measurement-Prediction Deviation

entry	group	euthanasia date	experimental weight (g)^a^	predicted weight (g)^b^	error (%)^c^
1	control	32	0.4	0.993	-
2	control	32	0.5	1.167	-
3	control	32	0.7	1.742	-
4	control	32	0.9	2.14	-
5	control	39	0.6	0.634	5.6
6	control	25	0.9	6.004	-
7	control	32	0.3	0.605	-
8	control	32	0.4	1.133	-
9	control	32	0.6	1.375	-
10	control	32	0.6	1.554	-
11	treatment	32	0.5	0.798	-
12	treatment	39	0.35	0.311	11.1
13	treatment	32	0.4	0.631	-
14	treatment	39	0.4	0.395	1.2
15	treatment	39	0.5	0.703	40.6
16	treatment	39	0.3	0.328	9.4
17	treatment	32	0.4	0.495	-
18	treatment	32	0.4	0.638	-

a Measured at euthanasia date.

b At day 39.

c Prediction error was computed as the absolute relative error: |experimental–predicted|/experimental × 100.

Predicted tumor weights on day 39 were 1.40 ± 0.59 g (mean ± SD) for controls and 0.52 ± 0.19 g for the treatment group, with hybrid experimental-predicted groups showing significant differences (p-value = 0.003, Cohen’s *d* = 1.069). This predictive capability facilitates standardization of tumor weights across all animals within the same study, even when euthanasia occurs on different dates due to humane end point criteria.

These analytical approaches are implemented in the freely available ViVo web platform (gcanudo-barreras.github.io/ViVo-Platform/), providing accessible computational workflows that enable direct application of exponential modeling, TGR matrix analysis and tumor weight predictive and standardization capabilities without specialized expertise requirements.

## Discussion

The ViVo framework demonstrates that mathematical modeling can substantially enhance statistical power while reducing animal requirements in preclinical oncology. The mechanistic advantage becomes particularly pronounced in small-sample scenarios, where conventional analyses often fail to detect genuine treatment effects. Our systematic evaluation reveals that exponential modeling maintains superior statistical performance across all tested sample sizes, achieving 90% power with *n* = 7,7 groups and 100% power above, compared to 61.5% maximum for conventional analysis with larger cohorts. This enhancement enables approximately 40% reduction in animal usage while maintaining robust detection capabilities, as demonstrated by the Daskalakis data set analysis where comparable statistical outcomes required substantially fewer animals.

The introduction of Tumor Growth Rate (TGR) matrices represents a methodological advance, transforming discrete measurements into comprehensive temporal maps of treatment dynamics. Our analysis revealed three distinct therapeutic phases: Early Response, Extended Efficacy, and Long-Term Response that remained invisible to conventional end point analysis. Notably, the observed kinetic differences between cell lines align with established literature, with 4T1 cells characterized as rapidly proliferating (https://www.atcc.org/products/crl-2539-luc2)^18^ and MDA-MB-231 cells as comparatively indolent (https://www.atcc.org/products/htb-26).^19^ The resulting 5-fold quantitative differentiation validates that the framework captures genuine biological kinetics rather than mathematical artifacts, providing confidence in the ability of the method to discriminate meaningful therapeutic effects from experimental noise.

This temporal granularity provides quantitative insights into treatment kinetics, revealing that therapies with similar overall efficacy may exhibit markedly different response patterns. The systematic identification of peak efficacy windows has direct implications for optimizing dosing schedules and designing rational combination therapies based on drug action kinetics.

### Methodological Positioning and Applicability

Compared with alternative mathematical approaches, exponential modeling offers an optimal balance between biological relevance and practical applicability for early stage tumor dynamics. While logistic and Gompertz models provide theoretical advantages for late-stage progression, they require extensive data sets and prolonged observation periods to capture critical parameters such as carrying capacity and inflection points.^1,2^ These requirements often conflict with standard preclinical protocols,^20,21^ which typically span 2–4 weeks and focus on proliferation-dominated phases where exponential assumptions remain valid.^2,11^

The exponential framework becomes particularly advantageous compared with modern AI-based approaches, which rely heavily on large, high-quality data sets for pattern generalization.^22^ In sample-limited settings, parameter-rich models suffer from reduced identifiability and compromised robustness.^23^ Our approach addresses this challenge by extracting biologically meaningful parameters from limited data, while maintaining mathematical rigor through goodness-of-fit thresholds and systematic quality control.

A critical aspect of successful implementation is the recognition of appropriate application boundaries. The exponential model accurately describes tumor dynamics during proliferation-dominated phases but becomes less reliable when angiogenic constraints and nutrient depletion alter the growth kinetics. Objective indicators of model breakdown, including systematic residual patterns and *R* ^2^ values falling below 0.8, serve as quality control thresholds, ensuring analytical reliability within well-defined operating ranges.

### Limitations and Critical Assessment

Several important limitations should be acknowledged when using this framework. The exponential growth assumption fundamentally restricts applicability to early stage tumor development, typically within the first 2–4 weeks of preclinical studies. Beyond this window, more complex growth dynamics emerge that require alternative modeling approaches. Additionally, the quality filtering process, while necessary for parameter reliability, can exclude animals with poor model fits, potentially introducing a selection bias that may not reflect true treatment responses.

Mathematical modeling faces intrinsic statistical limitations when applied to extremely small groups (*n* ≤ 4). Even with optimal model performance, such sample sizes constrain the reliability of parameter estimates due to fundamental statistical principles rather than methodological limitations. While our approach improves robustness in limited-sample contexts, thoughtful experimental design remains essential for ensuring valid and interpretable results.

The temporal analysis provided by TGR matrices, while offering enhanced resolution, requires careful interpretation to avoid false discoveries arising from multiple comparisons. Although we systematically evaluated 78 temporal intervals compared to 13 conventional time points, this increased analytical resolution must be balanced against appropriate statistical corrections to maintain type I error control. While the complete TGR matrix generates numerous interval comparisons, we argue against blanket correction for three reasons: **(1)** each interval addresses a distinct biological question about temporal treatment dynamics; **(2)** overlapping intervals violate the independence assumption of standard corrections; and **(3)** the exploratory nature of temporal pattern discovery favors sensitivity over specificity, consistent with established recommendations for discovery-phase research.^24,25^ Nevertheless, we provide Bonferroni and Benjamini-Hochberg corrected values for the subset of diagonal intervals that are statistically independent. Notably, TGR analysis demonstrates superior robustness under correction compared to conventional approaches: in the 4T1-Luc2 model, 100% of significant diagonal intervals survive both Bonferroni and Benjamini-Hochberg corrections (Supporting Information Table S7), versus only 50% of significant daily time points (Supporting Information Table S4).

Furthermore, the biological interpretation of short-term growth rate fluctuations requires careful consideration of the measurement precision and potential artifacts.

### Impact and Practical Applications

The alignment of this methodology with the 3Rs principles represents perhaps its most significant contribution to preclinical research. Our demonstration that mathematical modeling can achieve superior statistical performance with smaller sample sizes suggests the potential for meaningful animal reduction across preclinical oncology. Analysis of the Daskalakis data set indicates that exponential modeling achieves 70% statistical power using 12 animals while conventional analysis maintains 61.5% power with 18 animals, representing nearly 40% reduction in animal usage while improving analytical precision.

TGR analysis provides a promising tool for early detection of therapeutic response, determination of optimal treatment windows, and detection of early resistance signals. The systematic identification and removal of outliers, combined with standardized data quality assessment, address a critical challenge in preclinical studies where inconsistent data handling can compromise reproducibility. The predictive capabilities demonstrated through tumor weight standardization offer immediate practical benefits for addressing humane end point requirements. This application alone may justify implementation in laboratories struggling with data interpretation across heterogeneous sacrifice dates. The freely available ViVo platform embodies these principles, providing automated quality control and interactive analysis tools that are accessible to researchers without computational expertise.

## Conclusions

This framework demonstrates that mathematical modeling can substantially enhance the statistical power in preclinical tumor studies while reducing animal usage by up to 40%. By converting conventional measurements into growth rate parameters and enabling temporal pattern analysis through TGR matrices, the approach addresses fundamental challenges in small-sample preclinical research, where biological variability often masks genuine treatment effects.

The structured temporal data foundation provided by TGR matrices creates natural integration points for machine learning applications in precision oncology, positioning the framework as a potential bridge between conventional preclinical assessment and emerging computational approaches in personalized cancer therapy. As precision medicine strategies gain prominence in clinical oncology, the need for standardized preclinical evaluation methodologies that extract the maximum information from limited samples becomes increasingly critical.

While our current validation encompasses multiple cell lines and implantation sites, broader validation across diverse tumor models, particularly patient-derived xenograft (PDX) systems, remains essential for establishing methodological robustness. Several limitations should be acknowledged, including the exponential growth assumption that may not capture all tumor growth dynamics, and the *R* ^2^ thresholds, which should be adjusted judiciously to avoid introducing selection bias. Integration with existing laboratory information management systems and regulatory submission pathways will facilitate adoption across research institutions and pharmaceutical development programs.

The ViVo framework ultimately represents a convergence of mathematical rigor, practical accessibility, and ethical responsibility in preclinical research. By enhancing the analytical precision while reducing animal usage, this approach establishes a foundation for more efficient and responsible preclinical oncology research.

## Experimental Section

### Mathematical Framework

#### Exponential Growth Modeling

Growth rate parameters (*r*) were extracted through linear regression on log-transformed data. Quality assessment used the coefficient of determination (*R* ^2^) with configurable thresholds. We set *R* ^2^ > 0.8 as the default quality threshold, following established practice in tumor growth modeling where exponential models typically achieve *R* ^2^ values exceeding 0.8–0.9 when applied to appropriate early stage data. This threshold balances model selectivity with practical applicability, ensuring reliable parameter extraction, while avoiding overly restrictive filtering that could exclude biologically meaningful data.

Tumor volume was estimated using the standard ellipsoid formula (eq 4)­

4 $$V\approx \frac{LW^{2}}{2}$$

where *L* and *W* represent length and width measurements, respectively.

#### Model Assumptions and Validity Criteria

Model validity requires exponential growth conditions typically present during the first 2–4 weeks of preclinical studies. Application boundaries are defined by multiple criteria.

- *R* ^2^ threshold filtering (default > 0.8, configurable 0.0–1.0)
- Residual pattern analysis for systematic deviations from exponential behavior
- Temporal applicability boundaries based on study duration and growth phase
- Automatic flagging when growth patterns suggest model breakdown (systematic residual patterns, *R* ^2^ degradation below 0.8)

Indicators of model breakdown serve as objective boundaries for valid application, ensuring analytical reliability within well-defined operating ranges compatible with standard efficacy assessment protocols.

#### Tumor Growth Rate (TGR) Matrix Construction

For each temporal analysis, localized growth rates were calculated between all possible time point pairs using eq 3. This localized growth rate (*r*_*x*→*y*_) represents the instantaneous growth dynamics between specific time points *x* and *y*, enabling detection of temporal patterns invisible to whole-study analysis. When calculated for all possible time combinations, the localized growth rates form structured matrices where each element *r*_*x*→*y*_ describes a specific temporal interval with rows corresponding to starting time points and columns to ending time points.

#### Multiple Comparison Considerations

The TGR matrix generates *n*(*n* – 1)/2 pairwise interval comparisons from *n* time points. This large number of comparisons could raise concerns about Type I error inflation. We address this through a structured analytical framework that avoids indiscriminate correction for the following reasons:

First, each TGR interval tests a distinct biological hypothesis regarding treatment efficacy during a specific temporal window. The question “Does the drug suppress growth during days 0–3?” is biologically independent from “Does the drug suppress growth during days 7–11?”, even though both derive from the same experiment.

Second, off-diagonal TGR intervals are mathematically correlated because they share time point data (e.g., *r*_0→7_ contains day 0 data, same as *r*_0→3_). This correlation structure violates the independence assumption underlying both Bonferroni and Benjamini-Hochberg procedures.

Third, TGR analysis operates in a hypothesis-generating framework: discovering unobserved treatment effects and determining when these effects emerge and dissipate. As argued by Rothman and Perneger, routine application of multiple testing corrections in exploratory analyses may increase type II errors and obscure genuine biological patterns.^24,25^

Given these considerations, we report uncorrected p-values alongside effect sizes (Cohen’s d) as coprimary indicators of biological and statistical relevance. We acknowledge that this approach prioritizes discovery over strict control of false positives, which we consider appropriate given the exploratory nature of TGR analysis and the availability of effect sizes for interpretation. However, to accommodate different statistical philosophies, we additionally report: **(1)** Bonferroni-corrected p-values applied exclusively to diagonal elements *r*_*t*→*t*+1_, which represent consecutive nonoverlapping intervals that satisfy independence assumptions and **(2)** Benjamini-Hochberg FDR-adjusted q-values, also restricted to diagonal elements.

#### Automated Quality Control System

Before fitting the data into the exponential model, the Automated Quality Control System evaluates data homogeneity, searches for potential outliers, and analyzes them. The system employs interquartile range (IQR) analysis with configurable sensitivity thresholds that can be adjusted both automatically—based on sample size—or manually. The three sensitivity levels are.

- Ultra-conservative (*n* < 8)
- Conservative (*n* = 8–12)
- Moderate (*n* > 12)

#### Homogeneity Assessment

Before analysis, ViVo ensures data homogeneity using the coefficient of variation (CV) and a Homogeneity Quality Score that accounts for both CV and sample size. Assessment occurs twice: before and after outlier filtering to ensure transparency in data processing decisions.

For each animal, the baseline value is the first recorded measurement, with nonpositive baseline values excluded. The Homogeneity Quality Score combines a piecewise linear penalty on CV with multiplicative corrections for small-sample sizes. The base score uses two thresholds (ε = 15 for excellent, π = 30 for poor), with the formula (eq 5)­

5 $$score_{0}\left(CV\right)=\{\begin{array}{l} \max\left\{0,100-2\times \left(CV-\pi \right)\right\},\\ 95-\left(CV-\varepsilon \right),\\ 100, \end{array}\begin{array}{l} CV>\pi ,\\ \varepsilon <CV\leq \pi ,\\ CV\leq \varepsilon , \end{array}$$

A multiplicative adjustment factor accounts for small-sample uncertainty (eq 6)­

6 $$factor\;\left(n\right)=\left(n<5?s_{small}:1\right)\times \left(n<3?s_{very\;small}:1\right)$$

With the final score computed as (eq 7)­

7 $$score\;\left(CV,n\right)={score}_{0}\left(CV\right)\times factor\;\left(n\right)$$

#### Six-Criteria Outlier Detector

The platform implements systematic outlier detection through six criteria with severity-based classification (Supporting Information Table S9). The system employs interquartile range (IQR) analysis on log-transformed data with configurable sensitivity that automatically adjusts based on sample size:

Critical Severity.

- IMPOSSIBLE_VALUE: values ≤ 0
- EXTREME_GROWTH: excessive exponential growth (> 1000–5000%/day depending on sample size)
- EXTREME_DECLINE: excessive exponential decline (> 67–90%/day depending on sample size)

High Severity.

- INTRA_OUTLIER: outliers within individual animals using IQR analysis

Medium Severity.

- GROUP_OUTLIER: outliers compared to group median
- LAST_DAY_DROP: abrupt drops on the final measurement day

Sensitivity automatically adjusts with three levels: Ultra-Conservative (*n* < 5, 4.0× IQR multiplier), Conservative (*n* = 5–10, 3.0× multiplier), and Moderate (*n* > 10, 2.0× multiplier). For small samples, multiple indicators are required for outlier classification to prevent excessive data exclusion (Supporting Information Table S9).

### Dual Analysis Framework

Three complementary analysis approaches accommodate different data quality scenarios.

1 Complete Analysis: All animals and measurements included regardless of quality flags
2 Animal-Level Filtering: Entire animals excluded when containing selected anomalies
3 Point-Level Filtering: Individual problematic measurements excluded while retaining animals with ≥ 3 valid time points
This framework enables transparent assessment of outlier impact on study conclusions while keeping analytical rigor, allowing researchers to evaluate how quality control decisions affect their results.

### Statistical Analysis and Interactive Framework

#### Primary Statistical Comparisons

Growth rate comparisons between experimental groups employed Mann–Whitney *U* tests to handle small samples and avoid normality assumptions inherent to limited sample sizes.

#### Power Assessment Methodology

Statistical power evaluation employed systematic random subset selection from the original Daskalakis data set (*n* = 8,10). For each sample size scenario (*n* = 8,10 down to *n* = 3,3), 20 random subsets were generated to account for selection variability. For conventional analysis, statistical power was calculated as the percentage of study days significance (p-value < 0.05) averaged across all subsets with each scenario. For exponential modeling, power was calculated as the proportion of subsets detecting significant growth rate differences, with results categorized as binary outcomes (significant/nonsignificant) for each subset.

#### TGR Matrix Statistical Implementation

For TGR analysis, statistical comparisons utilize complete individual animal data, while displayed matrices show group-averaged values for visualization. The interactive analysis framework enables real-time statistical testing of specific temporal intervals through point-and-click selection, with automatic calculation of.

- Group medians and interquartile ranges for each temporal interval
- Mann–Whitney *U* test statistics and p-values for between-group comparisons
- Cohen’s d effect sizes with 95% confidence intervals

Effect sizes follow Cohen’s conventions: small (*d* = 0.2–0.5), medium (*d* = 0.5–0.8), and large (*d* ≥ 0.8), with comprehensive outputs including test statistics, significance determinations, and confidence intervals formatted for publication.

### Variance Reduction Quantification

Parameter variability was assessed using the relative standard error (RSE). The variance reduction mechanism follows error propagation principles (Supporting Information Note S1) where growth rate uncertainty scales quadratically with time (eq 2).

### Platform Implementation and Technical Specifications

#### System Requirements and Compatibility

The ViVo platform operates entirely in a browser using JavaScript ES6+, requiring no software installation or server-side processing. Compatible browsers include Chrome 60+, Firefox 55+, Safari 12+, and Edge 79+. Modern browser compatibility ensures broad accessibility across research environments while supporting the FileReader API and CSS Grid for optimal functionality.

#### Performance and Capacity Specifications

Processing time for typical preclinical studies (20 animals, 10 time points) averages < 5 s on standard computers. Platform performance validation confirms handling of data sets up to the maximum specifications without degradation in user experience or computational accuracy.

#### Data Processing Workflow

The complete analytical workflow follows the following automated steps.

1 File Upload and Validation: Structured CSV templates provided for data formatting, with automatic validation of required columns (Animal, Group, time point columns, optional Tumor_Weight)
2 Automated Quality Control Assessment: Automatic homogeneity evaluation and outlier detection using the Six-Criteria system
3 Model Fitting and Quality Filtering: Exponential parameter extraction with *R* ^2^ quality filtering and validity assessment
4 Analysis Selection: User choice between complete analysis, animal-level filtering, or point-level filtering approaches
5 Statistical Analysis: Automated growth rate comparisons and TGR matrix generation with interactive exploration capabilities
6 Results Export: Enhanced data files and comprehensive HTML reports with publication-ready formatting

### Data Export and Reproducibility Framework

#### Enhanced Data and Export Capabilities

ViVo generates augmented CSV files containing original measurements and calculated parameters.

- Initial tumor burden (*N*_0_) with relative standard errors and confidence intervals
- Growth rates (*r*) and doubling times [ln(2)/*r*] for each valid animal
- *R* ^2^ coefficients and exponential model quality metrics
- Outlier detection results with severity classifications and criteria explanations
- Predicted tumor weights for standardized time points when applicable

#### TGR Matrix Export Structure

Tumor Growth Rate matrices export as a structured CSV format with the following column organization.

- Animal_ID: Unique animal identifier for traceability
- Experimental_Group: Treatment group designation for statistical comparisons
- *r*_*x*→*y*_ columns: All possible temporal interval growth rates as feature vectors

This standardized export format facilitates seamless integration with external statistical software and machine learning pipelines while maintaining complete analytical transparency.

#### Automated Report Generation

The platform generates comprehensive HTML reports that integrate.

- Raw data quality assessment with homogeneity scores and outlier detection summaries
- Exponential growth curve visualizations with model fit assessments
- Statistical results with publication-ready formatting including confidence intervals and effect sizes
- TGR matrices with statistical comparisons
- Complete methodology documentation for reproducibility

Reports include comprehensive analysis summaries suitable for results presentation or publication of the Supporting Information.

### Validation Design

#### Cross-Platform Validation and Performance Metrics

Primary validation used the publicly available Daskalakis data set from subcutaneous xenograft studies. Additional validation encompassed four independent animal models across different cell line origins (human vs murine), growth kinetics (rapid vs indolent), implantation sites (mammary, pancreatic, subcutaneous), and immune status (athymic vs immunocompetent). This systematic approach ensures methodological applicability beyond the use of single experimental systems. Detailed model descriptions are in Supporting Information Note S3.

Performance assessment evaluated: (1) statistical power enhancement across sample sizes, (2) temporal pattern detection capability through TGR analysis, (3) tumor weight prediction accuracy for humane end point standardization, and (4) biological coherence validation through cross-platform kinetic consistency.

## Supplementary Material



## Data Availability

All source code for the ViVo platform is available at github.com/gcanudo-barreras/ViVo-Platform under the MIT License. The repository includes complete documentation, example data sets, and a live web application (gcanudo-barreras.github.io/ViVo-Platform/). Validation data sets from studies described in this work are included in the repository’s data folder. No restrictions apply to code access or usage.

## Notes

Exponential fitting of the Daskalakis data set; daily caliper and bioluminescence imaging (BLI) statistical comparisons for orthotopic and xenograft mouse tumor models (4T1-Luc2, MDA-MB-231-Luc2-GFP, MLK 3287-Luc); tumor growth rate (TGR) interval comparisons; parameters used to compute the homogeneity score; adaptive configurations for the six-criteria outlier detector across sensitivity levels; error propagation analysis for exponential growth models, including mathematical framework and derivation, results, theoretical implications, validation against empirical data, practical applications, and connection to statistical power enhancement; Cohen’s *d* effect size interpretation; and description of mouse models, including syngeneic and xenogeneic orthotopic models and subcutaneous syngeneic tumor models (PDF).

## Abbreviations Used:

BLI: bioluminescence imaging
CV: coefficient of variation
IQR: interquartile range
PDX: patient-derived xenograft
RSE: residual standard error
TGR: tumor growth rates.
