# Supplementary material for: ViVo: A Temporal Modeling Framework That Boosts Statistical Power and Minimizes Animal Usage
Source: J Med Chem. 2025 Dec 29;69(2):1724–34. doi: 10.1021/acs.jmedchem.5c03326 (PMC12833854; doi:10.1021/acs.jmedchem.5c03326)
Supplement: Supplementary file 1 [file jm5c03326_si_001.pdf]

Supporting Information

ViVo: A temporal modeling framework that boosts statistical power and minimizes animal usage

Guillermo Canudo-Barreras,*[,a] Eduardo Romanos,[b] Raquel P. Herrera,[a] M. Concepción Gimeno*[,a]

[a] Instituto de Síntesis Química y Catálisis Homogénea (ISQCH) CSIC-Universidad de Zaragoza, C/ Pedro Cerbuna 12, 50009 Zaragoza, Spain. E-mail: canudobarreras@unizar.es; gimeno@unizar.es

[b] Servicio de Imagen Médica y Fenotipado, Instituto Aragonés de Ciencias de la Salud, Centro de Investigación Biomédica de Aragón (CIBA), Avda. San Juan Bosco, 13, Planta D, E-50009 Zaragoza, Spain

Contents of Supporting Information

Supplementary Table S1: Exponential Fitting of the Daskalakis Dataset Data S2

Supplementary Table S2: Daily Caliper Comparisons of the Daskalakis Dataset Data (Raw Statistical Analysis) S3

Supplementary Table S3: Daily BLI Comparisons of the Orthotopic 4T-1-Luc2 Assay (Raw Statistical Analysis) S4

Supplementary Table S4: Daily Caliper Comparisons of the Xenograft 4T-1-Luc2 Assay (Raw Statistical Analysis) S4

Supplementary Table S5: Daily Caliper Comparisons of the MDA-MB-231-luc2-GFP Assay (Raw Statistical Analysis) S5

Supplementary Table S6: Daily BLI Comparisons of the MLK 3287-Luc Assay (Raw Statistical Analysis) S5

Supplementary Table S7: TGR Intervals Comparisons of the Xenograft 4T-1-Luc2 ....S6

Supplementary Table S8: Parameters used to compute the homogeneity score S7

Supplementary Table S9: Adaptative configurations for the Six-Criteria Outlier Detector based on the sensitivity levels. S7

Supplementary Note 1: Error Propagation Analysis for Exponential Growth Models ...S8

- Mathematical Framework S8
- Mathematical Derivation S8
- Result S8
- Theoretical Implications S8
- Validation Against Empirical Data S9
- Practical Applications S9
- Connection to Statistical Power Enhancement S9

Supplementary Note 2: Cohen's d Effect Size Interpretation S9

Supplementary Note 3: Mouse Models S10

- Syngeneic Orthotopic Model with 4T-1-Luc2 Cells S10
- Xenogeneic Orthotopic Model with MDA-MB-231-luc2-GFP Cells S10

Orthotopic Model with Syngeneic-like MLK 3287-Luc Cells S11
Subcutaneous Syngeneic Model with 4T-1-Luc2 Cells S11

Supplementary Table S1: Exponential Fitting of the Daskalakis Dataset Data

**Animal ID**	**Group**	**V_0 (mm^3)**	**r (day^{-1})**	**R^2 ***
101	Control	55.2	0.208	0.9532

102	Control	111.8	0.137	0.8856
103	Control	29.1	0.270	0.9525
104	Control	46.0	0.211	0.9663
105	Control	27.8	0.309	0.9516
106	Control	42.4	0.200	0.9625
107	Control	54.3	0.358	0.9524
108	Control	60.5	0.365	0.9276
201	Drug	49.7	0.183	0.9735
202	Drug	63.9	0.183	0.9075
203	Drug	23.8	0.161	0.8981
204	Drug	28.6	0.242	0.9376
205	Drug	36.6	0.186	0.9775
206	Drug	35.8	0.114	0.8088
207	Drug	43.9	0.039	**0.4700**
208	Drug	47.5	0.203	0.9407
209	Drug	38.9	0.160	0.9368
210	Drug	108.2	0.144	0.8688

* Animals that do not meet the R^2 quality criteria are excluded. $R^2 < 0.8$ (red), $R^2 < 0.9$ (blue).

Supplementary Table S2: Daily Caliper Comparisons of the Daskalakis Dataset Data (Raw Statistical Analysis)

Day	Control mean (mm^3)	Drug mean (mm^3)	p-value	p-Bonf*1	q-BH*2	Cohen's d	Significance*3
0	55.6	51.8	0.5338	1.0000	0.5338	0.323	ns
3	97.0	63.9	0.0205	0.2665	0.0683	1.089	*
4	146.8	75.1	0.0266	0.3458	0.0683	1.081	*
5	195.2	104.1	0.0343	0.4459	0.0683	0.907	*

6	251.2	141.4	0.1728	1.0000	0.1872	0.818	ns
7	550.6	217.3	0.0831	1.0000	0.1080	0.859	ns
10	832.9	269.9	0.0266	0.3458	0.0683	1.273	*
11	1041.5	323.8	0.0021	0.0273	0.0273	1.300	**†‡
12	754.8	413.0	0.0934	1.0000	0.1104	1.177	ns
13	911.5	473.0	0.0727	0.9451	0.1050	1.244	ns
14	1330.7	696.8	0.0420	0.5460	0.0683	1.283	*
17	1528.3	788.9	0.0400	0.5200	0.0683	1.506	*
18	1692.1	884.6	0.0240	0.3120	0.0683	1.678	*

*[1] Bonferroni-adjusted p-values (13 comparisons, threshold p < 0.0038). *[2] Benjamini-Hochberg FDR-adjusted q-values. *[3] Statistical significance assessed using Mann-Whitney U tests. p-Bonf: Bonferroni-adjusted p-values (13 comparisons, threshold p < 0.0038). q-BH: Benjamini-Hochberg FDR-adjusted q-values. Significance: *p < 0.05; **p < 0.01; ***p < 0.001 (uncorrected); †p-Bonf < 0.05. ‡q-BH < 0.05.

Supplementary Table S3: Daily BLI Comparisons of the Orthotopic 4T-1-Luc2 Assay (Raw Statistical Analysis)

Day	Control mean (p/s)	Drug mean (p/s)	p-value	Cohen's d	Significance*[1]
0	5.74E+08	3.75E+08	0.2786	0.703	ns
2	5.70E+08	6.45E+08	1	-0.204	ns
4	4.42E+09	2.54E+09	0.0148	1.145	*
7	4.03E+10	1.15E+10	0.0003	2.681	***

*[1] Statistical significance assessed using Mann-Whitney U tests with Cohen's d effect size calculations. Significance levels: *p<0.05, **p<0.01, ***p<0.001.

Supplementary Table S4: Daily Caliper Comparisons of the Xenograft 4T-1-Luc2 Assay (Raw Statistical Analysis)

Day	Control mean (mm^3)	Drug mean (mm^3)	p-value	p-Bonf*[1]	q-BH*[2]	Cohen's d	Significance*[3]
11	199.1	227.8	0.3416	1.0000	0.5124	-0.266	ns

14	348.8	307.7	0.9159	1.0000	0.9159	0.232	ns
18	533.4	346.6	0.4595	1.0000	0.5514	0.665	ns
21	832.1	403.9	0.0447	0.2682	0.1054	1.194	*
25	1089.0	524.2	0.0527	0.3162	0.1054	1.194	ns
32	1742.8	826.1	0.0049	0.0294	0.0294	1.381	**†‡
39*4	-	-	-	-	-	-	-

*1 Bonferroni-adjusted (6 comparisons, threshold p < 0.0083). *2 Benjamini-Hochberg FDR. *3 Statistical significance assessed using Mann-Whitney U tests with Cohen's d effect size calculations. Significance levels: *p<0.05, **p<0.01, ***p<0.001 (uncorrected). †p-Bonf < 0.05. ‡q-BH < 0.05. *4 No comparison was possible because the control group consisted of only one animal.

Supplementary Table S5: Daily Caliper Comparisons of the MDA-MB-231-luc2-GFP Assay (Raw Statistical Analysis)

Day	Control mean (mm^3)	Drug mean (mm^3)	p-value	Cohen's d	Significance*1
22	46.7	80.8	0.4225	-0.822	ns
25	102.3	147.0	0.4225	-0.580	ns
29	149.3	227.1	0.3939	-0.644	ns
32	194.2	356.9	0.2403	-0.870	ns

*1 Statistical significance assessed using Mann-Whitney U tests with Cohen's d effect size calculations. Significance levels: *p<0.05, **p<0.01, ***p<0.001.

Supplementary Table S6: Daily BLI Comparisons of the MLK 3287-Luc Assay (Raw Statistical Analysis)

Day	Control mean (p/s)	Drug mean (p/s)	p-value	Cohen's d	Significance*1
3	1.32E+08	1.32E+08	0.6232	0.000	ns
7	6.91E+08	4.50E+08	0.5708	0.410	ns
10	1.03E+09	6.93E+08	0.2123	0.572	ns
14	1.31E+09	8.01E+08	0.1620	0.748	ns

*1 Statistical significance assessed using Mann-Whitney U tests with Cohen's d effect size calculations. Significance levels: *p<0.05, **p<0.01, ***p<0.001.

Supplementary Table S7: TGR Intervals Comparisons of the Xenograft 4T-1-Luc2

$Day_x \rightarrow day_y$	Control mean (n)	Drug mean (n)	p-value	p-Bonf	q-BH	Cohen's d	Significance*1
11→14	0.168 (10)	0.102 (8)	0.004	0.020	0.010	1.498	**†‡
11→18	0.136 (10)	0.056 (8)	0.003	-	-	2.359	**
11→21	0.142 (10)	0.060 (8)	< 0.001	-	-	3.546	***
11→25	0.124 (10)	0.060 (8)	0.001	-	-	2.903	**
11→32	0.111 (9)	0.052 (8)	0.001	-	-	2.334	**
11→39	0.062 (1)*2	0.0523 (4)	-	-	-	-	-
14→18	0.084 (10)	0.033 (8)	0.006	0.030	0.010	1.510	**†‡
14→21	0.122 (10)	0.039 (8)	< 0.001	-	-	3.269	***
14→25	0.102 (10)	0.055 (8)	< 0.001	-	-	2.791	***
14→32	0.102 (9)	0.048 (8)	0.004	-	-	1.858	**
14→39	0.064 (1)*2	0.047 (4)	-	-	-	-	-
18→21	0.158 (10)	0.053 (8)	< 0.001	0.005	0.005	2.432	***†‡
18→25	0.118 (10)	0.056 (8)	0.004	-	-	1.972	**
18→32	0.099 (9)	0.046 (8)	0.009	0.030	0.010	1.534	**
18→39	0.066 (1)*2	0.0512 (4)	-	-	-	-	-
21→25	0.071 (10)	0.057 (8)	0.722	1.000	0.722	0.168	ns
21→32	0.077 (9)	0.047 (8)	0.149	-	-	0.662	ns
21→39	0.054 (1)*2	0.045 (4)	-	-	-	-	-
25→32	0.0796 (9)	0.0496 (8)	0.102	0.510	0.128	0.785	ns

25→39	0.064 (1)*[2]	0.044 (4)	-	-	-	-	-
32→39	0.048 (1)*[2]	0.026 (4)	-	-	-	-	-

*[1] Statistical significance assessed using Mann-Whitney U tests with Cohen's d effect size calculations. p-Bonf and q-BH corrections applied only to diagonal elements, which represent consecutive non-overlapping intervals satisfying statistical independence (5 comparisons, Bonferroni threshold p < 0.010). Off-diagonal elements share timepoint data and violate independence assumptions required for valid correction. Significance levels: *p<0.05, **p<0.01, ***p<0.001 (uncorrected). †p-Bonf < 0.05. ‡q-BH < 0.05 (diagonal only). *[2] No possible statistical comparison due to insufficient sample size.

Supplementary Table S8: Parameters used to compute the homogeneity score

Parameter	Symbol	Default value	Interpretation
Excellent threshold	ε	15	CV at or below this value receives maximal base score (100)
Good threshold	*Good*	25	Upper bound of the “good” interval (used for categorical labels)
Poor threshold	π	30	CV above this value receives an accelerated penalty (slope = −2)
Small-sample factor (applies if n<5)	s_{small}	0.8	Multiplies the base score for small groups (20% reduction)
Very-small-sample factor (applies if n<3)	$s_{verySmall}$	0.6	Additional multiplier for very small groups (40% reduction)

Supplementary Table S9: Adaptative configurations for the Six-Criteria Outlier Detector based on the sensitivity levels.

Sensitivity Level	Sample size	Max Growth (/day)	Max Decline (/day)	IQR Sensitivity	Notes
**Ultra-Conservative**	n < 5	5000%	90%	4.0	Requires multiple indicators for outlier detection
**Conservative**	n = 5-10	2000%	80%	3.0	Requires multiple indicators for outlier detection
**Moderate**	n > 10	1000%	67%	2.0	One indicator is sufficient for outlier detection

Supplementary Note 1: Error Propagation Analysis for Exponential Growth Models

Mathematical Framework

For an exponential growth function of the form: $N(t) = N_0 e^{rt}$

where N_0 is the initial tumor burden, r is the growth rate, and t is time, we derive the propagation of relative standard errors (RSE) from the model parameters to the predicted values.

Mathematical Derivation

Taking the natural logarithm of both sides:

$$\ln N(t) = \ln N_0 + rt$$

Differentiating with respect to the parameters:

$$dN / N = dN_0 / N_0 + t dr$$

This yields the relative error as:

$$\delta N / N = \delta N_0 / N_0 + t \delta r$$

where δN, δN_0, and δr represent the absolute errors in $N(t)$, N_0, and r, respectively.

Squaring both sides:

$$(\delta N / N)^2 = (\delta N_0 / N_0)^2 + 2t(\delta N_0 / N_0)(\delta r) + t^2(\delta r)^2$$

Assuming independence between parameter estimation errors (i.e., $E[(\delta N_0/N_0)(\delta r)] = 0$), the expected value becomes:

$$E\left[(\delta N / N)^2\right] = E\left[(\delta N_0 / N_0)^2\right] + t^2 E\left[(\delta r)^2\right]$$

Result

The relative standard error of the predicted tumor size is given by:

$$RSE(N) = \sqrt{\left[RSE^2(N_0) + t^2 \cdot RSE^2(r)\right]}$$

Theoretical Implications

This decomposition reveals that:

1. **Direct contribution**: Uncertainty in the initial size N_0 contributes directly to prediction uncertainty.
2. **Quadratic amplification**: Uncertainty in the growth rate (r) contributes quadratically with time, becoming the dominant source of error for extended studies.
3. **Variance reduction mechanism**: When comparing growth rates between treatment groups, the N_0 terms largely cancel out, leaving primarily the more precisely estimated r parameters.

Validation Against Empirical Data

Using the Daskalakis dataset, we validated this theoretical framework:

- **Predicted RSE reduction**: Formula suggests RSE(r) < RSE(V_0) for multi-timepoint data.
- **Empirical confirmation**: Observed RSE(r)/RSE(V_0) ratios of 0.46-0.56 consistent with theoretical predictions.
- **Time dependency**: Longer observation periods show greater variance reduction, as predicted by t^2 coefficient.

Practical Applications

This error propagation formula enables:

1. **Quantitative assessment** of prediction confidence intervals.
2. **Parameter importance ranking** identifies which parameter contributes most to model uncertainty.
3. **Experimental design optimization** suggests that precise estimation of tumor growth rate (r) is particularly critical for extended studies.
4. **Sample size planning** enables a priori estimation of expected RSE ratios for power calculations.

Connection to Statistical Power Enhancement

The mathematical framework explains why exponential modeling consistently outperforms size-based comparisons:

- **Volume/BLI comparisons**: Subject to full measurement error at each timepoint.
- **Growth rate comparisons**: Benefit from error averaging across multiple timepoints.
- **Temporal integration**: Mathematical constraints of exponential form reduce effective noise.
- **Between-group comparisons**: N_0 uncertainties largely cancel, emphasizing biologically relevant r differences.

This theoretical foundation validates the empirical power enhancement observed across all tested sample sizes and provides a quantitative framework for predicting method performance in future studies.

Supplementary Note 2: Cohen's d Effect Size Interpretation

Cohen's d quantifies the standardized difference between two group means, providing a scale-independent measure of effect magnitude that complements p-value significance testing. In our tumor growth rate analysis, Cohen's d was calculated as:

$$d = \frac{(mean_1 - mean_2)}{Sp}$$

Where,

$$\mathrm{Sp} = \sqrt{\left[\frac{\left((n_1-1)\times SD_1^2 + (n_2-1)\times SD_2^2\right)}{(n_1+n_2-2)}\right]}$$

Interpretation guidelines following Cohen's conventions:

- Small effect: d = 0.2-0.5 (subtle but meaningful difference)
- Medium effect: d = 0.5-0.8 (moderate practical significance)
- Large effect: d ≥ 0.8 (substantial practical significance)

In clinical contexts, effect sizes ≥0.8 indicate therapeutically relevant differences that are likely to translate into meaningful clinical outcomes. Our TGR analysis revealed predominantly large effect sizes (d > 1.0), indicating robust drug efficacy that extends beyond statistical significance to practical therapeutic relevance.

Cohen's d is particularly valuable for tumor growth studies because:

1. **Scale independence**: Allows comparison across different measurement units and timepoints.
2. **Clinical relevance**: Large effect sizes (d ≥ 0.8) suggest clinically meaningful tumor growth inhibition.
3. **Power analysis**: Facilitates sample size calculations for future studies.
4. **Meta-analysis compatibility**: Enables integration with broader literature syntheses.

The consistently large effect sizes observed across our therapeutic windows (d = 1.105-1.583) demonstrate not only statistical significance but substantial biological and potential clinical impact.

Supplementary Note 3: Mouse Models

Syngeneic Orthotopic Model with 4T-1-Luc2 Cells

A syngeneic orthotopic murine breast cancer model was established by implanting 4T-1-Luc2 cells (CRL-2539-LUC2™ / ATCC) into the mammary fat pad of athymic nude mice (Rj:ATHYM-*Foxn1*^{nu}-Janvier), which are maintained on a BALB/cAnNRj genetic background. Despite their immunodeficiency, these mice share sufficient genetic compatibility with the 4T-1 cell line to support a syngeneic tumor environment. The model was used to evaluate the efficacy of a compound during the initial 10 days of treatment (days 0, 2, 4, 7 and 10). Tumor progression was monitored by bioluminescence imaging (BLI). Mice were randomly assigned to two experimental groups: control and treatment (n=8,8).

Xenogeneic Orthotopic Model with MDA-MB-231-luc2-GFP Cells

A xenogeneic orthotopic model of human triple-negative breast cancer was established by implanting MDA-MB-231-luc2-GFP cells (Caliper Life Sciences) into the mammary fat pad of athymic nude mice. The model was used to evaluate the efficacy of a compound over a 32-day period. Tumor resection was performed on day 22 to assess metastatic progression. Tumor volume was estimated using caliper (Equation S1). The study included two experimental groups: control and treatment (n=6,6).

Orthotopic Model with Syngeneic-like MLK 3287-Luc Cells

An orthotopic murine pancreatic ductal adenocarcinoma (PDAC) model was established by implanting syngeneic-like MLK 3287-Luc cells into athymic nude mice to evaluate the efficacy of a compound over a 14-day treatment period. Tumor growth was measured by bioluminescence imaging (BLI). Animals were divided into two experimental groups: control and treatment (n=8,8).

The MLK 3287-Luc murine tumor-derived cell line was established in the laboratory of Dr. Carmen Guerra (https://orcid.org/0000-0002-3891-046X) from a genetically engineered mouse model generated by crossing multiple transgenic mouse lines carrying the following alleles: *ELA* (+/E), *TetO-Cre* (+/T), *Kras* (+/LSL-G12Vgeo), *p53* (lox/lox), *EGFR* (+/+), *ROSA26-LSL-YFP* (KI/KI), and *TgpCAG-LSL-KFP* (+/+).

Although athymic nude mice are immunodeficient, the murine origin and background of the implanted cells ensure a genetically compatible tumor microenvironment, making this model functionally syngeneic.

Subcutaneous Syngeneic Model with 4T-1-Luc2 Cells

A subcutaneous syngeneic murine breast cancer model was established by injecting 4T-1-Luc2 cells (CRL-2539-LUC2™ / ATCC) into immunocompetent mice (BALB/cByJRj-Janvier). The model was used to evaluate the efficacy of a compound from day 11 to day 39 of treatment (days 11, 14, 18, 21, 25, 32 and 39). Tumor volume was estimated using caliper (Equation S1). Mice were randomized into two groups: control and treatment (n=10,11).
